# Cyclic Vomiting Syndrome in 41 adults: the illness, the patients, and problems of management

**DOI:** 10.1186/1741-7015-3-20

**Published:** 2005-12-21

**Authors:** David R Fleisher, Blake Gornowicz, Kathleen Adams, Richard Burch, Edward J Feldman

**Affiliations:** 1 Department of Child Health, University of Missouri School of Medicine, Columbia, Missouri, USA; 2 Department of Medicine, University of Missouri Hospitals and Clinics, Columbia, Missouri, USA; 3 Cyclic Vomiting Syndrome Association, Milwaukee, Wisconsin, USA; 4 Department of Psychiatry, University of Missouri School of Medicine, Columbia, Missouri, USA; 5 Department of Medicine, Geffen School of Medicine at UCLA, Los Angeles, California, USA; 6 Department of Pediatrics, Geffen School of Medicine at UCLA, Los Angeles, California, USA

## Abstract

**Background:**

Cyclic Vomiting Syndrome (CVS) is a disorder characterized by recurrent, stereotypic episodes of incapacitating nausea, vomiting and other symptoms, separated by intervals of comparative wellness. This report describes the clinical features, co-morbidities and problems encountered in management of 41 adult patients who met the diagnostic criteria for CVS.

**Methods:**

This is a retrospective study of adults with CVS seen between 1994 and 2003. Follow-up data were obtained by mailed questionnaires.

**Results:**

Age of onset ranged from 2 to 49 years. The duration of CVS at the time of consultation ranged from less than 1 year to 49 years. CVS episodes were stereotypic in respect of their hours of onset, symptomatology and length. Ninety-three percent of patients had recognizable prodromes. Half of the patients experienced a constellation of symptoms consisting of CVS episodes, migraine diathesis, inter-episodic dyspeptic nausea and a history of panic attacks. Deterioration in the course of CVS is indicated by coalescence of episodes in time. The prognosis of CVS is favorable in the majority of patients.

**Conclusion:**

CVS is a disabling disorder affecting adults as well as children. Because its occurrence in adults is little known, patients experience delayed or mis-diagnosis and ineffectual, sometimes inappropriately invasive management.

## Background

Cyclic Vomiting Syndrome (CVS) consists of recurrent, stereotypic episodes of incapacitating nausea and vomiting lasting hours to days and separated by symptom-free intervals, which typically last weeks or months [1-5].

**Figure 1:**
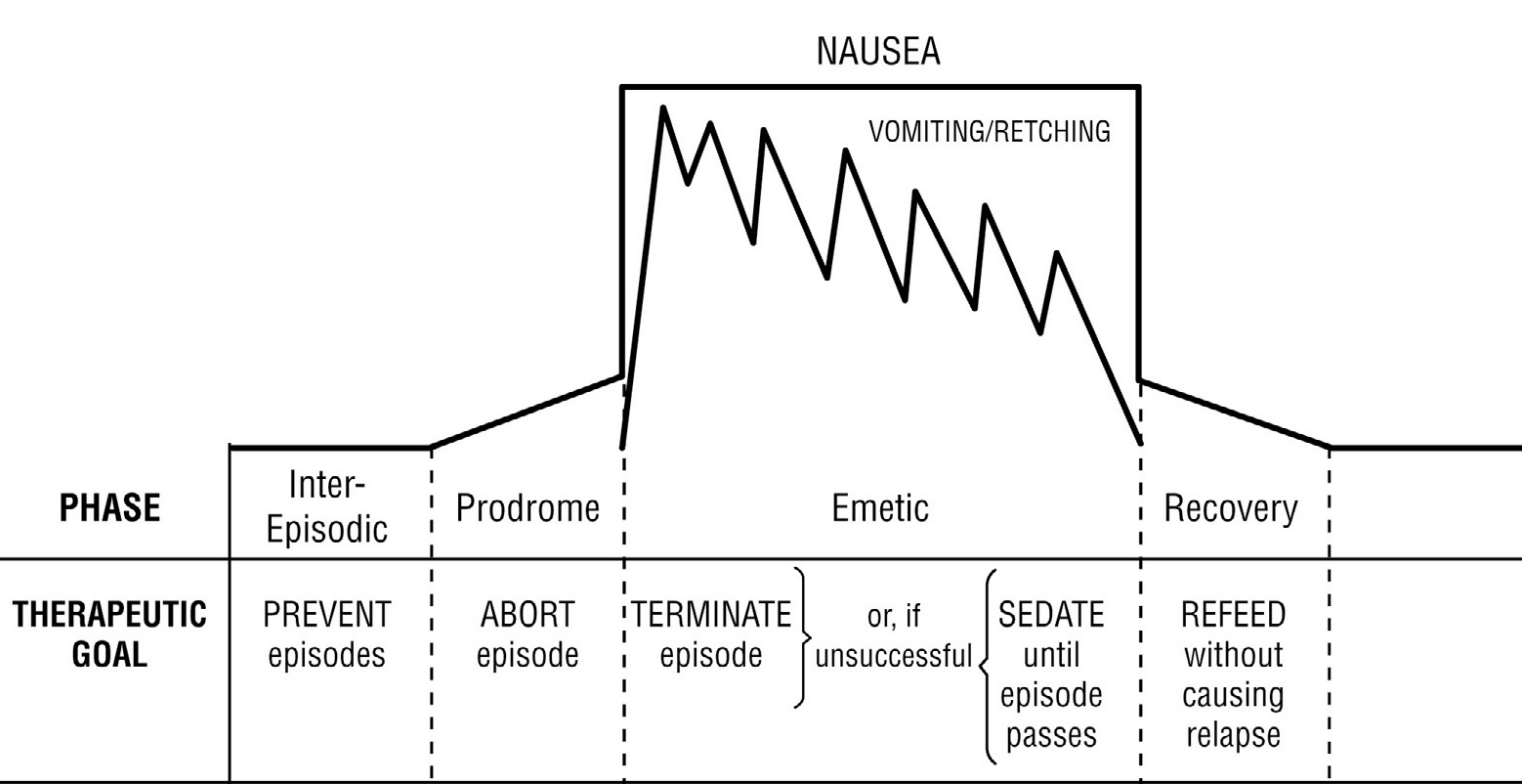
Schematic representation of the four phases of Cyclic Vomiting Syndrome and their therapeutic goals.

The illness has four phases [6]: the *inter-episodic phase*, during which the patient is relatively symptom-free; the *prodrome*, which begins when the patient begins to sense the approach of an episode, has nausea of varying intensity, but is still able to retain oral medications; the *emetic phase*, characterized by intense, persistent nausea, vomiting and other symptoms; and the *recovery phase*, which begins with the subsidence of nausea and ends when hunger, tolerance of oral intake and vigor return to normal (Figure 1). Episodes typically recur 6–12 times per year [1,3]. CVS is considered to be a functional disorder [7]. Some investigators have suggested that it is a manifestation of migraine diathesis [1,8-10].

CVS was first described in the English literature in 1882 by Samuel Gee who reported a series of nine children ranging in age from 4 to 8 years [10]. Thereafter, CVS was considered to be a pediatric disorder, and the absence of characteristic physical, laboratory or radiological markers for CVS resulted in its poor recognition by physicians who practice adult medicine. Cyclic Vomiting Syndrome has been reported in adults with onset ranging from childhood to middle age [12-14]. CVS in adults has been characterized as similar to CVS in children [15]. However, naturalistic observation of patients with CVS made during personalized continuity of care indicates that adults often present features and management challenges not typical of pediatric patients. Moreover, the impression is gained that the pattern of episodes in untreated CVS tends to remain more or less constant with respect to inter-episodic duration, duration of the emetic phase and associated symptoms[15]. By contrast, we found, and herein describe, the phenomenon of episode coalescence, which characterizes deterioration in the course of the illness. Although the high prevalence of migraine diathesis has been noted for more than 70 years [16], co-morbid anxiety, panic attacks and inter-episodic dyspeptic nausea are additional features of CVS in many adult patients. These features of CVS and their possible synergy in cyclic vomiting attacks have not been addressed previously.

We present a retrospective study of 41 consecutive adult patients selected from a cohort of 237 children and adults with CVS. Our purpose is to improve recognition of this frequently missed diagnosis by further describing the features of the disorder, the characteristics of patients afflicted with it, and problems encountered in its management.

## Methods

Forty-one adult patients were seen by one of us (DRF) between 1994 and 2003 with complaints compatible with the diagnostic criteria for CVS, namely, recurrent, self-limited, stereotypic episodes of intractable nausea and vomiting with no identifiable organic cause, separated by intervals of comparative well-being [2]. Clinical data were recorded on a standardized form to facilitate later retrospective analysis. Three hours were scheduled for each patient so that his/her illness, including psychological, social and developmental histories, could be explored phenomenologically [17] within the framework of the bio-psycho-social model of clinical practice [7,18-20]. Each patient was physically examined and a plan of management was negotiated [21-23]. Data for this report were extracted from patients' charts, which in some cases were insufficient for analysis of every feature described in our results; therefore, the sizes of databases from which statistics were derived vary. Follow-up data were collected by mailed questionnaires supplemented when necessary by telephone contact. They contained three questions: "Is your CVS better, worse or the same as it was at the time of our initial consultation?", "What made a difference, if any?", and, "About how many episodes have you had in the past 12 months and how long have they usually lasted?".

This study was approved by the Institutional Review Board of the University of Missouri – Columbia.

## Results

### Clinical characteristics of the syndrome

Forty-one adults (24 men and 17 women) presented at between 20 and 64 years of age (average, 34 years; median, 34 yrs) for evaluation of recurrent vomiting. The *age at onset *of the first episode ranged from 2 to 49 years (mean, 21 yrs; median 20 yrs).

The *duration *of the CVS at the time of consultation ranged from less than 1 year to 43 years (mean, 12 yrs; median, 7 yrs.).

The *severity *of CVS at the time of the consultation was deemed mild if it did not interfere with patients' ability to attend work or school; moderate, if employment or school enrollment were in jeopardy; and severe if the amount of time they were incapacitated by cyclic vomiting equaled or exceeded the amount of time they were well. Of 39 patients, 3 had mild, 17 had moderate, and 19 had severe CVS. Seven had been treated in emergency departments and as in-patients more than 100 times each. Four patients lost peripheral venous access and required central venous catheters. Thirteen (32%) were completely disabled and required financial support.

#### Stereotypy of episodes

**Figure 2:**
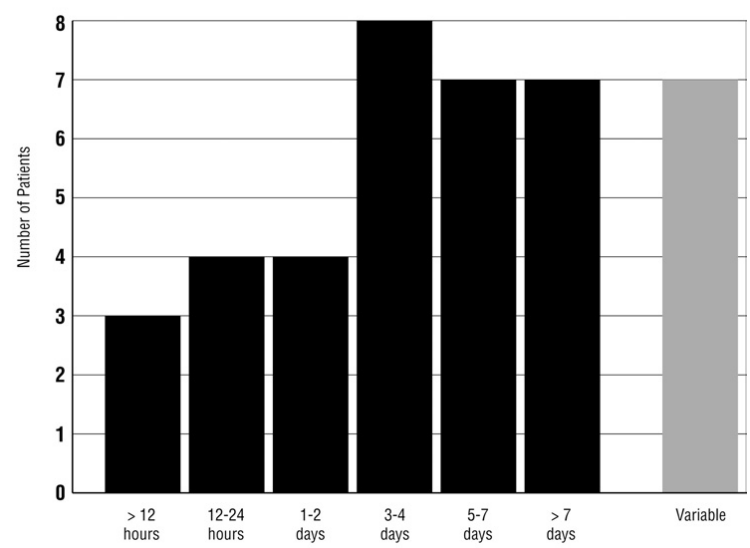
Duration of typical cyclic vomiting episodes in 39 patients.

**Figure 3:**
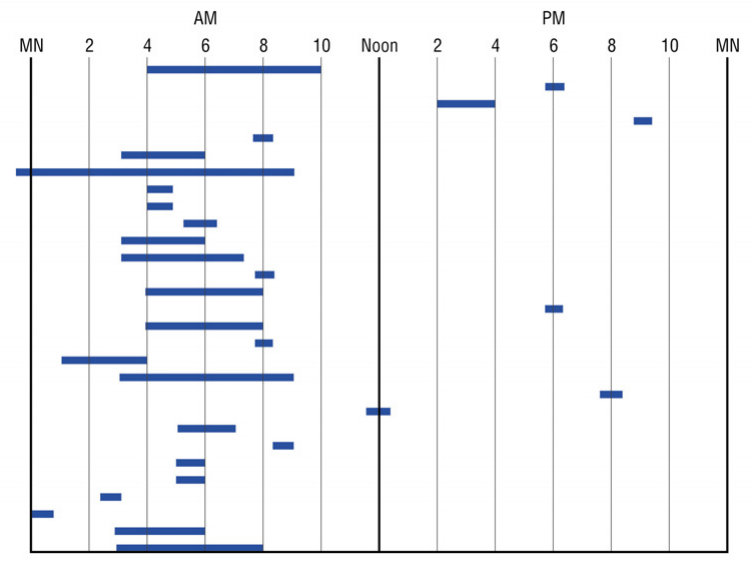
The ranges of times of onset of cyclic vomiting episodes in 29 adults who reported typical hours of onset. Morning hours predominate.

CVS episodes tend to be similar in *duration *and symptomatology over months or years [1,24]. Eighty-five percent of 39 patients had attacks of fairly uniform length and 15% had attacks of varying lengths (Figure 2). Typical *hours of onset *[1] in 29 patients are depicted in Figure 3. Most patients' episodes began between midnight and noon. Seven additional patients included four who reported "mornings," one who reported "afternoons or evenings," and two who reported that episodes began at any time during the day or night. Data were insufficient in five patients.

#### Triggering factors

Sixty-three percent of patients (16 men and 10 women) identified conditions that seemed to trigger the onset of cyclic vomiting episodes. The most frequent triggers reported by women were: menstrual periods in 7, noxious stress in 7, pleasant excitement in 5 and fatigue in 3. The commonest triggers in men were: noxious stress in 11, pleasant excitement in 8 and infections in 4.

#### The prodromal, emetic and recovery phases

The *prodrome *of a cyclic vomiting episode is important because it allows for attempts at aborting the emetic phase. Thirty-eight patients (93%) had recognizable prodromes. The remaining three patients were unable to identify a prodromal phase because they were already in the emetic phase when it woke them from sleep. The commonest prodromal symptoms in our patients are listed in descending order of frequency in Table 1.

**Table 1:** Symptoms reported during the prodromal phase of CVS

**Number of Patients Reporting**	**Symptoms During Prodrome**
23	Nausea
10	Sweating
9	Epigastric pain or "pressure"
9	Fatigue or weakness
8	Feeling hot and/or chilly
6	Cramping urge to defecate
5	Abdominal pain
5	Shivering or shakiness
5	Insomnia or restless, interrupted sleep
4	Aversion for food
4	Pounding or irregular heartbeat
3	Irritability
3	"Panic feelings"
2	Intense thirst
2	Feelings of depersonalization
2	Headache
2	Increased salivation
2	Dyspnea
2	A sensation of abdominal bloating
2	Inability to burp
2	Excessive burping
2	Inability to effect the desired passage of stool or flatus
2	Loose stooling
2	Epigastric burning
1	Paresthesias
1	Light headedness

The *emetic phase *was characterized by persistence of many prodromal symptoms plus severe, persistent nausea and vomiting. The maximum frequency of vomiting and retching ranged from once every 2 hours to 20 times per hour (average, 8.5 times per hour). The vomitus often contained blood and bile. Persistent, often severe abdominal pain occurred in 58% of patients. Low grade fever (100–102°F) was reported in 10 (25%) and neutrophilia without band-emia was documented in 9 (22%) of patients. Tachycardia with hypertension, which developed during episodes and subsided near the end of the emetic phase, was recorded in 9 patients.

The intense distress experienced by patients during the emetic phase of CVS prompted behaviors that were in some cases mistaken to be psychotic or bulemic. Many patients who were normally pleasant and cooperative became irritable, demanding and unable to think clearly or give an accurate history. Intense thirst (perhaps even while not dehydrated) was evident in 13 patients. It prompted sometimes bizarre efforts to access water; in two hospitalized patients, the interdiction of oral intake caused them to drink surreptitiously from toilets in the privacy of their locked bathrooms. Nineteen patients engaged in "guzzle-and-vomit" behavior. They repeatedly drank large amounts of liquid and then made themselves vomit. (One patient disposed of 14 liters in one day.) Patients reported that filling their stomachs diluted the irritants in their vomitus and lessened oro-pharyngeal pain during emesis. They also reported transiently decreased nausea following rapid evacuation of the stomach and that this self-induced, comfort-seeking maneuver was easier when the stomach was full rather than empty. Twenty-three of 41 patients (56%) took prolonged baths or showers several times a day during cyclic vomiting episodes. They reported that contact with water lessened the intensity of nausea, but only while they were in the tub or shower. The partial relief ceased as soon as they got out of the water. Hot water was used by all but one patient who preferred cold water.

The typical length of the *recovery phase *was reported by 22 patients and ranged from minutes to as much as ten days.

#### The worsening of Cyclic Vomiting Syndrome

The deterioration of CVS took the form of near- or complete coalescence of episodes. A near-coalescence occurs when episodes recur with increasing frequency. (The duration of the increasingly frequent episodes may be the same, shorter or longer than pre-coalescent episodes.) Patients who experience complete coalescence are sick more days than they are well and, in some cases, sick continuously for weeks at a time. Of 33 patients with episodes that had been fairly widely spaced, 21 experienced near-coalescences and six of those went on to have complete coalescences with persistent nausea and vomiting for as long as three weeks.

Previous medical records were available for 38 patients. The accumulated numbers of studies abstracted from records of our cohort are listed in Table 2. None revealed organic etiologies for their vomiting.

**Table 2:** Previous diagnostic studies reported in 41 adults with CVS

***Diagnostic Study***	**Number**
Esophago-Gastro-Duodenoscopies	62
Upper GI barium studies (with or without small bowel follow through)	44
Abdominal/pelvic ultrasonographies	43
Abdominal CT scans	33
Colonoscopies	24
Radionuclide Gastric Emptying Study	20
Urine porphyrins	16
Barium enemas	13
Hepatobiliary scans	10
H. pylori antibody assays	10
Cranial MRI's	9
Laparoscopies	5
Sinus radiographies	2
Endoscopic Retrograde Cholangio-Pancreatographies	1
Urine organic acid assays	1

**Total studies indicative of organic etiologies for cyclic vomiting**	**0**

Sixteen patients underwent 17 surgical attempts to cure their recurrent vomiting. The procedures are listed in Table 3. None resulted in improvement.

**Table 3:** Previous surgeries to relieve CVS in 41 adults

**Surgical Procedures**	**Number**
Cholecystectomies	10
Appendectomies	2
Exploratory laparotomy	1
Pyloroplasty	1
Gastrostomy and jejunostomy	1
Fundoplication	1
Hysterectomy	1

**Successful results**	**0**

### Characteristics of patients with CVS

CVS is considered to be a manifestation of migraine diathesis [10,25-27]. Of the 40 patients with sufficient data, 28 (70%) experienced migraine headaches during or between episodes. A diagnostic criterion of migraine is its familial occurrence [26-28]. Twenty-three (57%) of our patients had first and/or second-degree relatives with migraine headache or its variants. Of these, the family history was matrilineal in 19 (83%), patrilineal in 3 (13%), indeterminate (sibling only) in 1 (4%) and bilateral in none (29).

#### Psychiatric aspects of adult CVS

Clinically significant anxiety is common in children with CVS [30,31] and seems to be a predisposing factor to cyclic vomiting episodes [1,16,32-34]. Seven (17%) of our adult patients reported *anticipatory anxiety *as being an important triggering factor. Their fear of the next episode predisposed the onset of the next episode. The severity of previous episodes seemed to condition the severity of their anticipatory anxiety [35].

Histories of physical and/or emotional and/or sexual abuse during childhood were elicited from 18 (44%) of our adult patients. Twenty-nine (70%) of our adult patients had been previously diagnosed with, or had features characteristic of, one or more of the following: anxiety disorders, mood disorders, and alcoholism and/or drug abuse (not including non-recreational use of cannabis during episodes).

**Figure 4:**
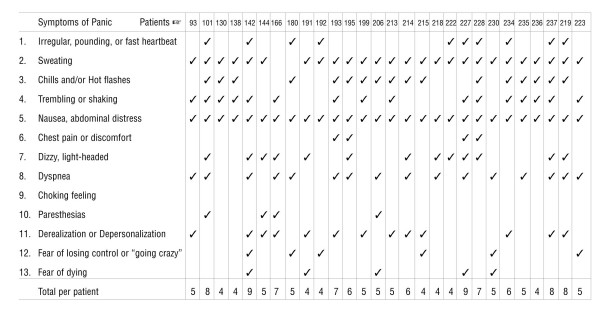
Panic symptoms in 21 men and 7 women during prodromal and emetic phases of cyclic vomiting episodes. The abrupt onset of intense fear or discomfort reaching a peak within 10 minutes, accompanied by four or more of the 13 symptoms listed on the left, are the diagnostic criteria for panic attacks [35].

Symptoms of panic attack were surprisingly prevalent in adult patients. The criterion for diagnosis of panic attack [35] is the occurrence of four or more of the thirteen symptoms listed on the left side of Figure 4. Twenty-eight (68%) or our adult patients (20 men and 7 women) had four or more panic symptoms during the prodromal and emetic phases of most or all their cyclic vomiting episodes.

#### Anxiety and patterns of nausea

Pfau and Li [37] analyzed a series of 106 children with recurrent vomiting and found two patterns: cyclic and chronic. The cyclic pattern, exemplified by CVS, consisted of episodes of continuous nausea and high intensity vomiting lasting hours or days and separated by several weeks of wellness. The chronic pattern, exemplified by the indolent nausea of peptic ulcer or inflammation of the upper GI tract, consisted of almost daily bouts of low intensity nausea and vomiting.

By contrast, 26 (63%) of our adult patients had a pattern of nausea and vomiting that was both cyclic and chronic. In addition to their cyclic vomiting episodes of high intensity nausea and vomiting, they had low intensity nausea and abdominal discomfort between episodes that was indistinguishable from functional dyspepsia [38]. Inter-episodic dyspeptic nausea occurred on arising in the morning or other times of the day and was predisposed by emotional stress or excitement [39-41]. The nausea lasted from a few minutes to a few hours. It did not always result in vomiting or prevent going to work or school.

**Figure 5:**
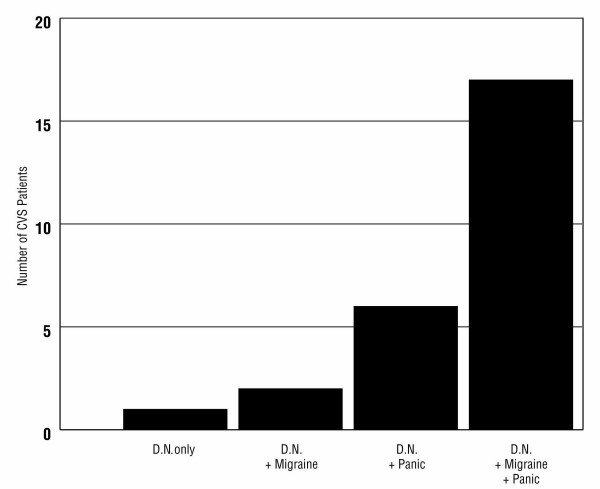
Twenty six CVS patients (63%) experienced dyspeptic nausea between episodes. Dyspeptic patients were more likely to have migraine diathesis and/or a history of panic attacks. Almost two-thirds of dyspeptic CVS patients had both migraine and panic.

A search for features associated with inter-episodic dyspeptic nausea showed that it was more common in patients with migraine diathesis and/or a history of panic attacks. Almost two-thirds of CVS patients with dyspeptic nausea had both migraine and panic attacks as co-morbid features (Figure 5).

### Follow-up

Follow-up questionnaires were sent 1 to 10 years after the initial consultation (average, 4.1 years, median, 3 years). Of our 41 patients, 37 (90%) responded. Of the 4 non-responders, one had died of systemic lupus, three could not be located and one refused to participate.

In answering the first question ("Are your symptoms better, worse or the same as when we first consulted?"), 32 (86%) indicated "better," none indicated "worse," and 5 (14%) indicated "the same." The responses to the second question ("What do you think made a difference?"), included: use of effective medications (mentioned by 22 patients); having an effective protocol to abort or treat the emetic phase promptly (9 mentions); learning the diagnosis of CVS (9 mentions); a good relationship with a physician knowledgeable about CVS (8 mentions); learning how to control "stress" (6 mentions); and six miscellaneous responses (having a baby, improved diet, prayer, an alternate diagnosis of celiac disease, moving to a less toxic environment, and "not sure").

**Figure 6:**
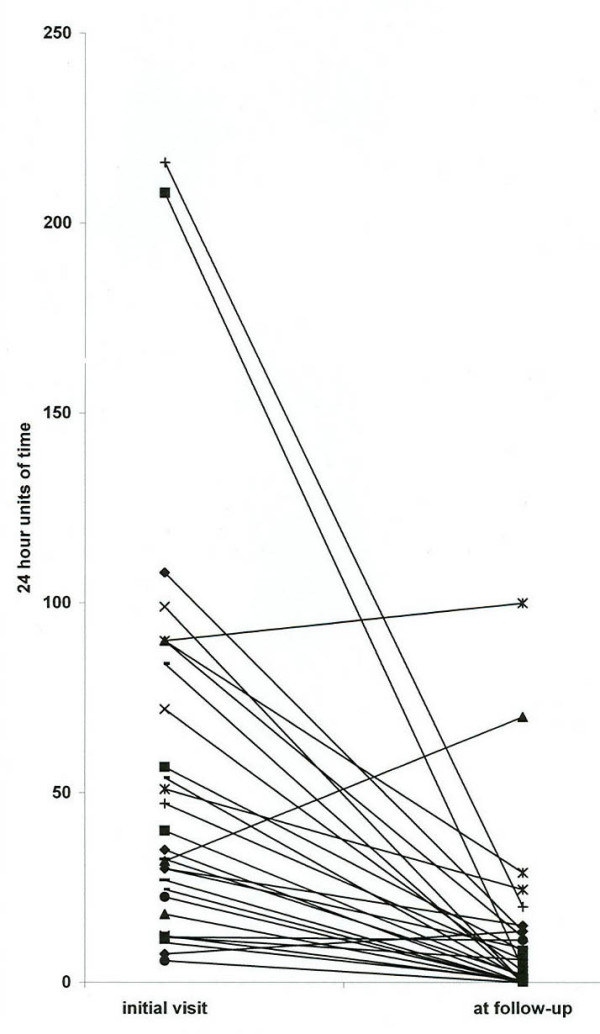
Comparison of the cumulative duration of cyclic vomiting episodes per year at the time of the initial consultation and at the time of follow-up in 29 patients. Data points are the products of the estimated number of episodes per year multiplied by the average duration of episodes and are expressed as 24-hour units of time. The intervals between initial consultations and follow-up ranged from 1 to 10 years and averaged 3.8 years.

Answers to the third question ("About how many episodes have you had in the past year and how long have they usually lasted?") allowed for a comparison of each patient's illness during the year prior to the initial consultation and during the year prior to the follow-up questionnaire, as shown in Figure 6. The intervals between the initial visits and follow-up ranged from 1 to 10 years, averaging 3.8 years. Otherwise, these data are, at best, semi-quantitative because they are based on each patient's subjective estimates at two points in time. The data points in Figure 6 were derived as the products of the number of attacks and the usual duration of attacks expressed as 24-hour units of time per year. Sufficient data were available from 30 patients. By this measure, 27 improved and 3 became worse. The improvement in the 27 ranged from 6.7% to 100%, averaging 84% less time being sick. The deterioration in 3 ranged from 11% to 119%, averaging 70% more time being sick. (A 4^th ^patient whose illness had deteriorated was omitted from Figure 6 because he had developed a persistently coalescent pattern of symptoms caused by panic disorder, which had become refractory to psychiatric management. Therefore, the deterioration was severe, but not readily quantifiable.)

## Discussion

The pattern of nausea and vomiting in CVS is unmistakably different from other vomiting disorders. Rumination and bulimia are accompanied by little or no nausea. The nausea related to reflux disease or achalasia is comparatively mild and transient. By contrast, the nausea of a cyclic vomiting episode is accompanied by dramatic autonomic dysfunction, diminished muscle tone and altered mental state causing total incapacitation that persists for the duration of the emetic phase.

The pathogenesis of cyclic vomiting episodes is obscure; their signs and symptoms mimic other disorders. For example, vomiting, abdominal pain, stress-induced neutrophilia [42] and elevated serum amylase of salivary origin may mimic pancreatitis, peptic ulcer, appendicitis or pyelonephritis. Nausea of any etiology [43-45] may cause myoelectrical abnormalities and delayed gastric emptying suggestive of organic gastroparesis. The periodicity of recurrences, with or without hypertension, mimics porphyria, pheochromocytoma, abdominal epilepsy, intermittent small bowel obstruction or endometriosis. The presence of gallstones or the finding of impaired gallbladder emptying [46] in patients whose cyclic vomiting episodes feature nausea, vomiting and epigastric pain, had prompted cholecystectomies that failed to relieve cyclic vomiting symptoms [14]. Although many organic diseases present a cyclic or recurrent *pattern *of vomiting [47], the Cyclic Vomiting *Syndrome *is, by definition, primarily a disorder of function which appears to originate in the central nervous system, not the abdomen [10,41,48].

### The significance of panic attacks in adults with CVS

Pediatric patients with CVS have higher rates of anxiety than children generally [1,10]. Panic disorder is uncommon in pre-adolescent children [49], but the emotional traumas that cause childhood anxiety have also been experienced during the childhoods of many adult patients who, by virtue of their longevity, may have lived long enough for panic to have evolved [50,51].

Conditions that are co-morbid with migraine include mood disorders, panic disorder and irritable bowel syndrome [52-57]. The psycho-social histories in about half of our cohort suggest that conditions co-morbid with migraine are likely to be found in adults with CVS when studied prospectively. Such studies may also show that the nausea experienced between episodes by 63% of our cohort is a psycho-physiological reaction to persistent anxiety [43].

Panic attacks, which can occur in patients with panic disorder as well as other anxiety disorders, usually last 5 to 20 minutes [58]. Some CVS patients who are anxious may experience brief panic attacks during the inter-episodic phase of their illness. However, in two thirds of our adult CVS patients, panic attacks triggered cyclic vomiting episodes during which they not only suffered intense nausea and vomiting, but also panic symptoms that persisted for hours or days throughout part or all of their cyclic vomiting episodes. The signs and symptoms of such prolonged panic often resemble the "adrenergic storm" in patients with pheochromocytoma during discharges of catacholamines, specifically, diaphoresis, tachycardia, palpitations, hypertension, nausea and vomiting, pallor, flushing, chest discomfort, abdominal pain and apprehensiveness [59]. Stress induces hypothalamic release of Corticotropin Releasing Factor (CRF) [60]. CRF activates the locus ceruleus, causing increased adrenergic tone. It also stimulates inhibitory fibers in the Dorsal Motor Nucleus of the vagus, thereby suppressing gastric motility, a concomitant, if not the immediate cause, of nausea [60,61].

### Cyclic Vomiting Syndrome and migraine

The report of a series of 214 children with idiopathic CVS by Li et al. [10] suggests that pediatric CVS patients can be subdivided into two groups: migraine-associated CVS (82%) and non-migraine associated CVS (18%). The first subgroup had, or subsequently developed, migraine headaches and/or had family histories of migraine; they were more likely to benefit from anti-migraine therapy. The second subgroup had no migrainous features and responded poorly to anti-migraine drugs. The implication that could be drawn from this study is that these are different kinds of CVS with different pathogeneses and managements.

Our experience with adult patients suggests an alternative, less dichotomized hypothesis. There may be at least two routes to a cyclic vomiting attack. One involves whatever creates attacks of migraine or migraine equivalents in patients with migraine diathesesis [29,53,62,64,65]. The other involves whatever creates anxiety attacks in patients with anxiety disorders [51,66-73]. The population of CVS patients might thus be composed of patients who are "purely" migrainous, devoid of pathological anxiety; patients with severe anxiety and panic attacks but no migrainous features; and patients who have elements of both migraine and anxiety that predispose to cyclic vomiting episodes. The efficacy of prophylactic and ameliorative medications might therefore depend on the appropriateness of the match-up between the therapeutic agents administered (e.g. anti-migraine, anti-anxiety or both) and the pathogenic factors in play [52]. Suffice to say that CVS and/or migraine headaches need to be seen within the context of their co-morbidities and that patients with these disorders need to be managed with their biology, psychology and social milieu in mind [20,23].

### The complication of attack coalescence

The emergence of coalescent patterns of attacks cause patients and clinicians to fear that CVS is becoming uncontrollable. A useful hypothesis for the pathogenesis of coalescence is based on the effects of anxiety and dread. While some CVS patients suffer pre-morbid anxiety disorders of various kinds that predispose them to nausea, in others, the disruption of normal development, the inability to be gainfully employed or pursue an education, and the strains that CVS imposes on family life create frustration, anxiety and depression that is secondary to the burden of illness. Many patients, particularly those whose episodes are especially painful and long, dread the episode-to-come so much that the level of autonomic hyperactivity in the interval between attacks approaches the level during an actual attack itself. In such cases, the distinction between cyclic vomiting episodes and intervening periods of wellbeing may become unclear [58].

### Management

The concurrence of inter-episodic dyspeptic nausea, migraine and panic in two-thirds of this cohort of adult CVS patients suggests that these dysautonomic phenomena share pathways or mechanisms within the central and autonomic nervous systems [62,63]. The aim of treatment is to help the patient regain the feeling of being in control rather than defenseless against recurrences of misery. Treatment of CVS should be prompt and as minimally stressful as possible [74]. CVS may take months or decades to overcome. The patient and physician together learn what exacerbates and what controls symptoms. Patients with CVS need a physician who is familiar with the syndrome, accessible, responsive, collaborative [22], non-judgmental, caring and has a rational, organized approach to management [75,76]. Long waits in emergency rooms, encounters with caregivers who are unfamiliar with CVS, receiving implausible diagnoses, the repetition of unrewarding diagnostic procedures, and stop-gap intravenous hydration followed by being sent home still sick, are common experiences that reinforce patients' feelings of being out of control of an illness that no one understands or can treat. Such experiences seem to promote coalescent patterns of attacks.

There is as yet no evidence-based treatment of CVS [77]. Although principles of management are helpful, optimal management for any patient is likely to differ from that of any other patient [52]. Treatment is applied according to the phase the patient is in at the time of presentation. The therapeutic goals during the inter-episodic phase include migraine *prophylaxis*, e.g. amitriptyline [17,78], propranalol [79], cyproheptadine [78,80] and others [81,82] if migraine diathesis is suspected. Conditions that pre-dispose to or trigger episodes (e.g. anxiety, chronic infections, pre-menstrual syndrome [83]) need to be identified and treated.

The therapeutic goal during the prodromal phase is to *abort *the onset of vomiting. To this end, discern the prodrome's constituent symptoms and attempt to relieve each as quickly as possible before vomiting begins. For example, ondansetron, lorazepam or alprazolam, and ibuprofen or oxycodone, might be taken for their anti-nausea, anxiolytic and analgesic effects. If prodromal symptoms subside as the patient rests, and if the remission lasts, the emetic phase would have been successfully aborted.

The therapeutic goals in the emetic phase are the prevention of dehydration, renal insufficiency, electrolyte depletion, tetany, hematemesis, and secretion of inappropriate anti-diuretic hormone (SIADH) [84,85]. If the emetic phase cannot be aborted it should be treated without delay, preferably within an hour of onset. "Watchful waiting" or long waits in treatment facilities are counter-therapeutic. Try to *terminate *the episode by bolus correction of dehydration, if it exists, followed by intravenous fluid maintenance, intravenous H2 receptor blocker or proton pump inhibitor and intravenous administration of anxiolytic and anti-emetic agents. If nausea fails to clear, or if it subsides only to return within a short time, the only way to relieve the patient's distress is by *sedation*, which should be continued until the emetic phase passes. Deep sleep stops vomiting that originates in the brain and makes the patient insensible to nausea and other distress [48]. When patients know they can get relief promptly, anticipatory anxiety subsides and, in time, coalescent trends abate [35]. Preferred sedative drugs are non-addictive and non-emetogenic. Tachycardia with high blood pressure is indicative of a hyper-adrenergic state, but tachycardia with low blood pressure suggests hypovolemia, which should be corrected before administration of sedative agents with potential vasodilatory side-effects. Urine specific gravity determinations can be used for surveillance of possible SIADH. Hematemesis may be due to prolapse gastropathy [86,87]. Although hematemesis of this type seldom causes serious blood loss, it does not preclude bleeding from erosive esophagitis or Mallory-Weiss tears. Intravenous opiates are necessary for pain control in some patients. However, they should be made aware of opiates' anxiolytic effects and the potential for dependence when opiates are used indiscriminately for anxiety as well as abdominal pain. Symptoms of opiate withdrawal, e.g. irritability, restlessness, nausea, cramps, insomnia, anxiety [88], may be misinterpreted as those of panic and/or the prodromal and emetic phases of CVS.

Keep the patient's room dimly lit and quiet. As much as possible, use brief intervals of spontaneous wakefulness for measurements of vital signs and urine specimen collections so as to minimize interruptions of sleep. Agitation, water seeking and "guzzle-and-vomit" behaviors are far better managed with effective sedation than by attempting to prevent the wakeful patient from engaging in them.

The recovery phase tends to be prolonged when inadequate management of the emetic phase has permitted severe fluid and electrolyte deficits or weight loss. Some patients are able to tolerate a regular diet as soon as their nausea subsides. Others may suffer a relapse if they take more than clear liquids initially. Prophylactic medications should be resumed as soon as the patient is able to do so comfortably.

Follow-up data suggest that the prognosis for CVS is generally favorable. Our clinical impression is that the severity and duration of CVS are influenced by the tractability of the co-morbidities that predispose to episodes [20]. Although assiduous management of its physical and psychological aspects seems to benefit patients, the relationship between its course and treatment modalities remains to be clarified by future prospective studies. Such research will require clear descriptions of the range of clinical features of adult CVS, a goal towards which this work aims to contribute.

## Conclusion

CVS affects patients of all ages. CVS is a disorder in which the patient's emotional wellbeing strongly influences the success of management. Frustration and despair are in themselves pathogenic; feelings of optimism and gaining-control are therapeutic. Pharmacological agents may be potent, but their efficacy depends to a large extent on the therapeutic quality of the doctor-patient relationship in which they are deployed [22,23,89,90].

## Competing interests

The author(s) declare that they have no competing interests.

## Authors' contributions

David R. Fleisher: Principal clinician and author

Blake Gornowicz: Co-author and collaborator in organizing and extracting chart data

Kathleen Adams: Co-author and collector of follow-up data

Richard Burch: Co-author and psychiatric clinician and consultant

Edward J. Feldman: Co-author, gastroenterologist and principal editor

## Pre-publication history

The pre-publication history for this paper can be accessed here:


